# Identifying associations between sample characteristics, symptoms, and self‐efficacy differences in adult patients with rare tumors of the central nervous system who participated in a novel web‐based natural history study

**DOI:** 10.1002/cam4.70017

**Published:** 2024-08-05

**Authors:** Alvina Acquaye‐Mallory, Elizabeth Vera, Anna Choi, Kathleen Wall, Mark R. Gilbert, Terri S. Armstrong

**Affiliations:** ^1^ Neuro‐Oncology Branch, Center for Cancer Research National Cancer Institute, National Institutes of Health Bethesda Maryland USA

**Keywords:** cancer prevention, medical oncology, psychosocial studies, QOL

## Abstract

**Objective:**

High self‐efficacy is associated with improved self‐care and reduced symptoms in cancer patients but has not been fully interrogated in adults with central nervous system (CNS) cancers. We aimed to identify the relationship between self‐efficacy levels in managing emotions (SEMEM) and social interactions (SEMSI) by examining sample characteristics and symptom burden.

**Methods:**

Sample characteristics and patient‐reported outcome (PRO) measures addressing self‐efficacy (PROMIS SEMEM & SEMSI) and symptom burden (MDASI BT or SP) were collected in a novel web‐based study of 158 adult patients diagnosed with rare CNS tumors.

**Results:**

The sample was predominantly female (73%), diagnosed with an ependymoma (66%), and had a median age of 45 (19–75). Low SEMEM was associated with a longer duration of symptoms before surgery (*r* = −0.26) and female gender (92%) among brain tumor (BT) participants and in spinal cord tumors (SCT), those with lower education (*r* = 0.29). Reporting low SEMSI was associated with being married (42%), lower education (*r* = 0.22), and a prolonged time with symptoms before surgery (*r* = 0.29) in those with BTs, with no associations identified in SCT. More severe mood‐related interference (including mood, enjoyment of life, and relationship with others) was associated with lower SEMEM among both locations (*r* = −0.61 brain, *r* = −0.28 spine) and SEMSI in BT participants (*r* = −0.54).

**Conclusions:**

Low self‐efficacy was linked to a prolonged time between symptom onset and initial surgery, education, gender, and marital status and was associated with higher mood‐related interference. Understanding characteristics associated with low self‐efficacy underscores a need for future studies to tailor interventions that enhance self‐efficacy.

## 1 BACKGROUND

Although all central nervous system (CNS) tumors are considered rare, a more uncommon set of rarer CNS tumors, accounting for 4400 new cases per year have been identified.^1^ Patients with these tumors are challenged by a scarcity of supportive and standard‐of‐care treatments, resources, and a need for knowledgeable healthcare professionals.^2^, ^3^ Often diagnosed at a young age,^4^ these tumors can be low grade^3^ and have long‐term effects on patients.^5^ Limited reports to date underscore that those diagnosed with these rare CNS tumors can be just as symptomatic as more common and more malignant CNS tumors,^3^ have functional impairments,^6^ and experience changes to their work status due to their diagnosis.^6^ Resources for symptom management can often be insufficient for patients. Schaefer and colleagues^7^ report the lack of consistent self‐management strategies available to address primary brain tumor patients' symptoms throughout their care. A study of adolescents and young adults diagnosed with diverse cancers, including CNS tumors, reported that when given tools to understand symptom experience, improvements in confidence and self‐management behaviors were seen, which helped patients feel comfortable in symptom discussions with providers.^8^

An essential component of self‐management in disease‐related factors is patients' perceived self‐efficacy,^9^ or belief in one's ability to execute behaviors to attain a desired outcome,^10^ a vital attribute for adherence to treatment recommendations and effective symptom management.^11^, ^12^ Since self‐efficacy is an adaptable construct, identifying areas where patients need assistance building their self‐efficacy is essential.^13^ Furthermore, overcoming past obstacles can positively influence self‐efficacy beliefs^10^; therefore, collecting and tracking patients' symptom experiences over time is vital to understanding patients' self‐efficacy levels. Although no studies to our knowledge have been completed in those with rare CNS tumors, having a higher perceived sense of control or self‐efficacy in coping effectively with the adverse effects of a cancer diagnosis leads to positive outcomes, including improvements in depression,^14^, ^15^ self‐regulation,^8^, ^16^ reduced symptom distress,^17^, ^18^ and a higher quality of life.^19^, ^20^ Rare CNS tumors remain an understudied group, particularly concerning self‐efficacy. A meta‐analysis assessing the relationship between self‐efficacy, distress, and quality of life mainly included breast cancer patients, with no studies focusing primarily on glioma patients.^12^ The authors identified that patients reporting higher self‐efficacy coped better with adjustment to overwhelming situations and exhibited less anxiety.^12^ Similar findings were observed in a study of a small sample of glioma patients, where highly efficacious patients endorsed lower distress scores.^18^ Understanding factors associated with low self‐efficacy can inform the development of accurate screening tools and early interventions for patients lacking confidence.^13^, ^21^

To address the self‐efficacy in patients with rare CNS tumors and to better understand the patient experience, a novel, web‐based Natural History study was developed within the National Cancer Institute's Comprehensive Oncology Network Evaluating Rare CNS Tumors (NCI‐CONNECT) initiative that is focused on 12 rare CNS tumors. We hypothesized that a relationship between self‐efficacy levels and symptom burden and interference of symptoms with daily life exists and evaluated sample characteristics that can be associated with differences in self‐efficacy consistent with findings in other studies described above. Furthermore, we conducted comparisons in our findings to explore the impact of self‐efficacy, which can provide valuable insights on areas to address in this patient population in future research.

## 2 METHODS

The Institutional Review Board approved this study at the National Institutes of Health (ClinicalTrials.gov Identifier: NCT03251989, P.I. T. Armstrong) in August 2017. This study was performed in accordance with the ethical standards established by the US Federal Policy for the Protection of Human Subjects. Participants were recruited through various media sources, including social media and websites (Collaborative Ependymoma Research Network, Cancer.gov, NCI‐CONNECT). Eligibility criteria included: (1) 18 years of age or older with initial diagnosis occurring at any point in one's lifetime, (2) ability to sign consent and English speaking and (3) if they self‐identified with 1 of the 12 rare CNS diagnoses [atypical teratoid rhabdoid tumor (ATRT); brainstem and midline gliomas; choroid plexus tumors; ependymoma; high‐grade meningioma; gliomatosis cerebri; medulloblastoma; oligodendroglioma/anaplastic oligodendroglioma; pineal region tumors; pleomorphic xanthoastrocytoma/anaplastic pleomorphic xanthoastrocytoma; PNET (supratentorial embryonal tumor); primary CNS sarcoma/secondary CNS sarcoma (gliosarcoma)]. After completing an online enrollment form, a telephone consent was conducted. Once participants signed the informed consent form, they received a unique link to complete the rare CNS outcomes survey. The approximate time to complete was 50 min. Survey sections included demographic and social information data, tumor details (surgery, recurrence, and current treatment), and patient‐reported outcomes (PRO) measures related to symptom burden and self‐efficacy.

### 2.1 Instruments

#### 2.1.1 MD Anderson Symptom Inventory‐Brain Tumor (MDASI‐BT) and Spine Tumor (MDASI‐SP) modules

The MDASI is a PRO measure tailored to identify location‐specific symptom burden and interference with daily life functioning within the last 24 h in patients diagnosed with cancer. The instrument includes core symptoms common to most cancers and specific symptoms associated with tumor location and treatment throughout the illness trajectory. Based on the participant's tumor location, the MDASI‐Brain Tumor (MDASI‐BT) or the MDASI‐Spine Tumor (MDASI‐SP) modules were used in the study, consisting of 22 and 18 symptom items, respectively. Each symptom is rated on an 11‐point scale from 0 (not present) to 10 (as bad as you can imagine). How symptoms interfere with daily life are also measured, including activity‐related WAW including walking, general activity, work (including housework) and mood‐related (REM including relations with other people, enjoyment of life, and mood) items using the same rating scale from 0 (Did not interfere) to 10 (Interfered completely). A symptom rating ≥5, REM and WAW scores ≥2 were considered moderate–severe. For the objectives of this study, mood‐related symptoms represented as the affective symptom factor grouping and individual symptoms (fatigue, disturbed sleep, feeling distressed, feeling sad, and irritability) of the MDASI‐BT and the emotional factor (feeling distressed, feeling sad) of the MDASI‐SP were hypothesized to be associated with self‐efficacy and evaluated. The MDASI‐BT and MDASI‐SP have been validated for use in neuro‐oncology patients.^22^, ^23^

#### 2.1.2 PROMIS self‐efficacy for managing emotions and PROMIS self‐efficacy for managing social interactions

The PROMIS SEMEM is a four‐item instrument measuring confidence in regulating negative feelings, stress, discouragement, and disappointment. The PROMIS SEMSI is an eight‐item measure assessing confidence in social interactions and seeking help when necessary. Responses to items ranged from “I am not at all confident” to “I am very confident.” Raw scores are converted into *T*‐scores that show self‐efficacy levels. A *t*‐score of 50 represents the US general population (SD = 10), and cut‐off scores can be interpreted as follows: ≤40 = lower confidence, 50 = average, and ≥60 = greater confidence. These measures have been validated for adult patients with chronic conditions and have demonstrated correlations with other scales, including the Self‐efficacy for Managing Chronic Disease 6‐item scale (legacy scale) and the Global Health (physical & mental), Physical Function, Fatigue, Depression, and Anxiety PROMIS short forms.^24^

### 2.2 Statistical methods

Descriptive statistics were used to report frequencies, mean, and range of sample characteristics. Spearman rho correlations were used to identify the relationship between self‐efficacy t‐scores and mood‐related interference scores. Chi‐square tests of association, Fisher's exact test with OR, or Cramer's *V* as effect size, and point‐biserial correlations were used between self‐efficacy levels and interference severity, demographics and clinical characteristics, and symptom severity. Statistical analyses were conducted using IBM SPSS Statistics, Version 28.^25^

## 3 RESULTS

Table 1 highlights the demographic and clinical characteristics. A total of 158 (brain: *n* = 87, spine: *n* = 71) participants completed the PROMIS self‐efficacy measures. The sample had a median age of 45 (19–75). Participants were primarily female (73%) and married (65%), with a higher level of education. Most participants were diagnosed with an ependymoma (66%). Notably, 35% were on surveillance, and 37% of the sample had initial symptoms for a year or more before surgery.

**TABLE 1:** Sample characteristics.

	Total *N* = 158	
	*n*	%
Sex		
Female	115	73
Male	41	26
Non‐binary/Third gender	1	1
Missing	1	1
Race		
White	150	95
Other race	7	4
Missing	1	1
Hispanic/Latino		
Yes	7	4
Missing	1	1
Marital status		
Never married	24	15
Married	102	65
Divorced	16	10
Separated	8	5
Re‐married	3	2
Widowed	3	2
Missing	2	1
Education		
Some high school	3	2
High school graduate	7	4
Some college	32	20
College graduate	63	40
Any postgraduate work	52	33
8th grade or less	1	1
Missing	0	0
Diagnosis		
ATRT	2	1
Brainstem & Midline gliomas	2	1
Choroid plexus tumors	1	1
Ependymoma	104	66
Gliomatosis cerebri	1	1
Medulloblastoma	4	3
Oligodendrogliomas	21	13
Pineal region tumors	20	13
Pleomorphic xanthoastrocytomas	3	2
Treatment status		
Newly diagnosed	6	4
No treatment other than surgery	33	21
On treatment	24	15
Follow‐up without active treatment	53	34
Other	38	24
Missing	4	3
Time from symptoms to surgery		
No symptoms	9	6
<1 month	13	8
1–2 months	20	13
3–4 months	14	9
5–6 months	13	8
7–11 months	21	13
1–4 years	45	28
≥5 years	14	9
Missing	9	6
Recurrence		
Yes	27	17
Missing	12	8
Work status		
Not working	74	47
Working	82	52
Missing	2	1
Work changes		
None	55	35
Reduce hours	34	22
Switch jobs	4	3
Retrain	1	1
Stop working	50	32
Lost work due to tumor	11	7
Missing	3	2

### 3.1 Self‐efficacy scores

Table 2 summarizes the self‐efficacy scores from the PROMIS measures. None of the participants with a brain or spinal cord tumor (SCT) identified having greater confidence (score ≥60) in managing social interactions; less than a quarter had this greater confidence level in managing emotions. Among the sample, more participants with a brain tumor (BT) reported low confidence (score ≤40) in managing emotions (17% vs. 7%, *p* = 0.034, Cramer's *V* = 0.21) and. in social interactions (28% vs. 7%, *p* = 0.001, OR = 4.95) compared to those with a SCT. Additionally, this latter group showed higher confidence on SEMSI (*p* = 0.016, Hedge's *g* = 0.38), scoring 3.4 points higher than those with a BT.

**TABLE 2:** Summary of self‐efficacy (T‐scores) by tumor location.

	Brain	Spine	Brain	Spine
	Social interactions	Social interactions	Managing emotions	Managing emotions
*N*	87	70	87	71
Mean	47.5	51.0	48.9	50.1
SD	10.1	7.6	9.3	7.1
Minimum	25.2	31.2	29.5	33.7
Maximum	59.8	59	63.1	63.1
Percentiles				
25	38.8	44.7	42.2	45.5
50	49.4	50.0	48.4	49.7
75	59.8	59.8	56.7	54.2
Low	28%	7%	17%	7%
Average	72%	93%	63%	82%
High	0%	0%	20%	11%

### 3.2 Self‐efficacy and demographic and clinical characteristics

An association between self‐efficacy levels and sample characteristics among tumor locations (brain vs. spine cord) was identified in the sample and is presented in Table 3. The results of SEMEM showed married BT participants (82%) were more likely to endorse higher self‐efficacy (*p* = 0.030, Cramer's *V* = 0.24). In contrast, low self‐efficacy was associated with being a female BT participant (93%, *p* = 0.050, Cramer's *V* = 0.26) as well as experiencing a prolonged time with brain tumor symptoms before initial surgery (*r* = −0.26, *p* = 0.022, *n* = 80). Spine participants not on active treatment were more likely to report high SEMEM (p = 0.041, Cramer's *V* = 0.37), while low self‐efficacy was associated with lower education (*r* = 0.29, *p* = 0.014, *n* = 71). BT participants reporting low SEMSI were more likely to be married (42%, *p* = 0.004, Cramer's *V* = 0.37), and also a correlation was identified in those with lower educational levels (*r* = 0.22, *p* = 0.038, *n* = 87) and a longer time with presenting symptoms (*r* = −0.29, *p* = 0.009, *n* = 80). No associations with clinical or demographic factors were identified for SEMSI among SCT participants.

**TABLE 3:** Associations among sample characteristics and levels of self‐efficacy.

	Brain						Spine						Brain					Spine				
	SE: Managing emotions						SE: Managing emotions						SE: Social interactions					SE: Social interactions				
	*N*	Low	Avg	High	Sig	OR/Cramer's *V*	*N*	Low	Avg	High	Sig	OR/Cramer's *V*	*N*	Low	Avg	Sig	OR/Cramer's *V*	*N*	Low	Avg	Sig	OR/Cramer's *V*
**Gender**	86						70						86					69				
Female		93%	65%	56%	0.050	0.26		100%	81%	63%	0.251	—		83%	63%	0.076	—		100%	78%	0.575	—
Male		7%	35%	44%				0%	19%	38%				17%	37%				0%	22%		
**Race**	86						71											70				
White		93%	98%	94%	0.294	—		80%	98%	75%	0.018	0.36		96%	97%	1.000	—		100%	95%	1.000	—
Other race		7%	2%	6%				20%	2%	25%				4%	3%				0%	5%		
**Marital status**	85						71						85					70				
Never married		23%	18%	0%	0.030	0.24		20%	17%	0%	0.686	—		21%	13%	0.004	0.37		20%	15%	0.195	—
Married		38%	69%	82%				80%	64%	88%				42%	77%				40%	69%		
No longer married		38%	13%	18%				0%	19%	13%				38%	10%				40%	15%		
**Working**	87						69						87					68				
No		53%	49%	53%	1.000	—		20%	46%	38%	0.596	—		50%	51%	1.000	—		40%	43%	1.000	—
Yes		47%	51%	47%				80%	54%	63%				50%	49%				60%	57%		
**Work change**	86						69						86					68				
None		7%	39%	47%	0.060	—		60%	30%	63%	0.366	—		17%	42%	0.073	0.24		60%	35%	0.352	—
Changes but employed		40%	28%	12%				20%	25%	13%				38%	23%				0%	25%		
Stopped work		53%	33%	41%				20%	45%	25%				46%	35%				40%	40%		
**Treatment status**	85						69						85					69				
Newly diagnosed		7%	4%	6%	0.179	—		0%	3%	0%	0.041	0.37		8%	3%	0.245	0.24		0%	3%	0.766	—
No treatment		13%	25%	0%				0%	28%	33%				21%	16%				40%	25%		
On treatment		13%	26%	18%				0%	9%	0%				21%	23%				0%	8%		
Follow‐up		33%	25%	53%				0%	38%	67%				17%	38%				20%	39%		
Other		33%	21%	24%				100%	22%	0%				33%	20%				40%	25%		
**Recurrence**	77						69						77					68				
No		92%	82%	73%	0.512	—		80%	79%	100%	0.420	—		89%	80%	0.499	—		80%	81%	1.000	—
Yes		8%	18%	27%				20%	21%	0%				11%	20%				20%	19%		

### 3.3 Self‐efficacy and symptom severity

A relationship was identified between overall symptom burden and both self‐efficacy measures: SEMEM (brain: *r* = −0.54, *p* < 0.001; spine: (*r* = −0.25, *p* = 0.035)) and SEMSI (brain: *r* = −0.55, *p* < 0.001). Participants reporting severity in symptom burden were also found to report lower self‐efficacy. Self‐efficacy was also associated with specific types of symptoms, including moderate–severe mood‐related symptoms (affective factor grouping on the MDASI‐BT and emotional factor of the MDASI‐SP (Table 4)). BT participants who demonstrated low SEMEM also reported moderate–severe *fatigue* (73%, Cramer's *V* = 0.31), *disturbed sleep* (73%, Cramer's *V* = 0.38), *feeling distress* (80%, Cramer's *V* = 0.50), *feeling sad* (80%, Cramer's *V* = 0.53) and *irritability* (73%, Cramer's *V* = 0.49). Additionally, SCT participants who demonstrated low SEMEM also reported moderate–severe *feeling distress* (80%, Cramer's *V* = 0.33) or *feeling sad* (60%, Cramer's *V* = 0.31). BT participants who demonstrated low SEMSI were more likely to report specific moderate–severe symptoms, including *fatigue* (75%, OR = 4.3), *disturbed sleep* (75%, OR = 8.1), *feeling distress* (58%, OR = 4.9), *feeling sad* (61%, OR = 6.0) and *irritability* (58%, OR = 8.2).

**TABLE 4:** Associations among levels of self‐efficacy and severity of interference and symptoms.

	Brain						Spine					
	SE: Managing emotions						SE: Managing emotions					
	*N*	Low (%)	Avg (%)	High (%)	Sig.	OR/Cramer's *V*	*N*	Low (%)	Avg (%)	High (%)	Sig.	OR/Cramer's *V*
REM (MS)	87	93	64	18	<0.001	0.48	69	80	61	38	0.321	–
WAW (MS)	87	73	56	29	0.038	0.27	69	100	71	50	0.152	–
Fatigue (MS)	87	73	53	24	0.016	0.31						
Disturbed sleep (MS)	87	73	40	12	0.002	0.38						
Feeling distress (MS)	87	80	27	6	<0.001	0.50	69	80	23	38	0.018	0.33
Feeling sad (MS)	86	80	28	0	<0.001	0.53	69	60	14	25	0.036	0.31
Irritability (MS)	86	73	19	12	<0.001	0.49						

	SE: Social interactions						SE: Social interactions					
	*N*	Low (%)	Avg (%)		Sig.	OR	*N*	Low (%)	Avg (%)		Sig.	OR
REM (MS)	87	92	48		<0.001	12.1	68	75	58		0.638	–
WAW (MS)	87	79	44		0.004	4.75	68	50	72		0.575	–
Fatigue (MS)	87	75	41		0.008	4.3						
Disturbed sleep (MS)	87	75	27		<0.001	8.1						
Feeling distress (MS)	87	58	22		0.002	4.9	68	75	25		0.063	–
Feeling sad (MS)	86	61	21		0.001	6.0	68	25	17		0.549	–
Irritability (MS)	86	58	15		<0.001	8.2						

### 3.4 Self‐efficacy and MDASI‐BT/SP interference scales

The relationship between self‐efficacy measures and mood‐related and activity‐related interference of symptoms with daily life is presented in Table 4. Participants reporting severity in REM and WAW also reported lower self‐efficacy. The results showed a relationship between SEMEM, and mood‐related interference or REM (brain: *r* = −0.61, *p* < 0.001; spine (*r* = −0.28, *p* = 0.019)), and activity‐related interference or WAW (brain: *r* = −0.40, *p* < 0.001), and SEMSI and mood‐related interference or REM (*r* = −0.54, *p* < 0.001), and WAW (*r* = −0.43, *p* < 0.001) only for BT participants. Moderate–severe REM endorsed by BT participants was more common in those with low SEMEM (low (93%), average (64%), and high (18%)) (*p* < 0.001, Cramer's *V* = 0.48), but no associations were identified in SCT participants. Reporting of moderate–severe WAW was identified in BT participants endorsing low (73%), average (56%), and high (29%) SEMEM (*p* = 0.038, Cramer's *V* = 0.27), with no associations found in spine participants. As we previously reported, high confidence was not endorsed by the sample in managing social interactions, however, a relationship was found in BT participants, with a higher proportion of those reporting low (92%) and average (48%) self‐efficacy scores, endorsing moderate–severe REM (*p* < 0.001, OR = 12.1) and those reporting low (79%) and average (44%) self‐efficacy, reporting moderate–severe WAW (*p* = 0.004, OR = 4.75). No associations among spine tumor participants were identified between SEMSI and neither REM or WAW.

## 4 DISCUSSION

Studies focused on patients with rare CNS tumors are scarce; accrual remains a major challenge, as a small number of patients are diagnosed per year with care dispersed across multiple centers and providers.^1^, ^3^ Through social media outreach, our study successfully enrolled nine of the 12 identified rare CNS tumor types. Our findings demonstrated associations between self‐efficacy levels related to managing emotional distress, social interactions, symptom severity, and symptom‐related mood and activity‐related interference with daily life.

Low self‐efficacy was associated with lower education and a prolonged time between symptom onset and surgery in brain and spine participants. Numerous international studies have found a link between lower educational attainment and low self‐efficacy in coping with cancer‐related symptoms,^26^ self‐care,^27^ and overall perceived self‐competence.^28^ Interestingly, while prior studies did not identify the time since surgery as a significant variable associated with self‐efficacy, understanding the unique barriers faced by individuals with very rare cancers seeking help during health status changes can inform our perspective on patients' self‐efficacy as they obtain a diagnosis in addition to coping with their disease.^29^

Patients with CNS tumors have neurologic symptoms related to tumor location in the brain or spine, cognitive changes, and physical limitations that may necessitate home assistance. In evaluating SEMEM, female BT participants reported lower self‐efficacy. Conversely, high self‐efficacy was associated with being married or not being on active treatment. A study encompassing various cancer diagnoses found female participants reported lower self‐efficacy scores within a year of completing initial treatment, with the lowest scores related to managing fatigue and emotional distress; however, marital status was not associated with any level of self‐efficacy.^13^ Prior research has also demonstrated that confidence in managing emotions and maintaining daily activities predicts lower depressive symptoms^14^ in cancer survivors (primarily breast). Kenzik and colleagues reported that in older cancer survivors (≥ 65 years. old), regardless of how follow‐up care or treatment care plans were obtained, female sex, minorities, and those having at least one comorbidity exhibited lower confidence in managing cancer symptoms.^30^ Since a third of our patients were not on active treatment and considering that neuro‐oncology patients are often symptomatic after surgery and treatments,^31^ understanding the link between self‐efficacy, emotional distress, and survivorship care planning may be important.

Our results on SEMSI identified an association between being married (42%) and low self‐efficacy in BT participants, which is the opposite of what we found in SEMEM. While one might assume that having a spouse would be beneficial, the nature of the relationship can impact confidence levels. In head and neck cancer patients, a link between self‐efficacy and social control (a technique to influence behavior to encourage adherence) emerged.^32^ Spouses who employed positive social control (more supportive engagement) witnessed increased adherence to self‐care behaviors to manage symptoms.^32^ This positive adherence was associated with higher self‐efficacy and improved mood in patients compared to negative social control situations.^32^

Previous research has confirmed that individuals with more common CNS glial tumors report lower self‐efficacy in coping with symptoms, endorse a higher level of distress, and need more psychosocial support.^18^ In our sample, worsening activity or mood interference with daily life activities was associated with low self‐efficacy among BT participants on both measures and spine on the SEMEM instrument. Interestingly, depression often goes undetected in CNS patients,^33^ however it can occur in 20% of patients 8 months into their diagnosis and may impact long‐term survivors.^34^ As we recognize that self‐management is an essential factor in patients' self‐efficacy^9^ for managing symptoms, understanding the contributing factors can help tailor studies aimed at improving self‐efficacy and adherence, particularly in areas related to mood disturbance.

### 4.1 Study limitations

Limitations of this study include the cross‐sectional design, as understanding the impact of self‐efficacy early in the diagnosis and throughout the illness trajectory may guide when and how researchers can intervene. While the study was broad and encompassed both brain and SCT patients, exploring differences among more common tumor types would enhance our understanding of the overall experience in neuro‐oncology patients. This study employed two short‐form versions (4a & 8a) to assess patients' self‐efficacy levels. Utilizing the longer short form (8a) for both measures may have been beneficial, as it facilitates improved scoring and precise measurement of the specific construct.^35^ Additionally, focusing on only two constructs to identify patients' self‐efficacy may have limited our ability to identify unmet needs in this population. Utilizing a comprehensive self‐efficacy measure that incorporates various domains could strengthen our findings.

### 4.2 Clinical implications

To our knowledge, this is one of the first studies examining self‐efficacy in patients with CNS tumors. Our study highlights the importance of identifying self‐efficacy levels and recognizing the impact on this patient population. Differences in self‐efficacy support the need to target specific groups to pilot interventions such as CBT and social support interventions that have been shown to improve or maintain self‐efficacy in other cancer populations.^12^, ^36^ However, evaluating these interventions in vulnerable individuals with rare CNS tumors may require addressing both the impact of neurologic disease and significant neurologic and cognitive symptoms associated with having cancer.

## 5 CONCLUSION

Understanding self‐efficacy levels is essential to providing adequate care for patients with rare CNS tumors who struggle to feel confident in managing emotions and social interactions. We found an association between patients' self‐efficacy and sample characteristics (gender, marital status, treatment status, race, prolonged time until surgery). Additionally, confidence in managing emotions and social interactions worsened with severe mood and activity‐related interference in daily life. Our findings highlight the need for further research in self‐efficacy within this patient population to gain insight into how we address psychosocial and symptom management needs. Comparable results were identified regarding patients' self‐efficacy levels in the PROMIS instruments and the comprehensive self‐efficacy measures utilized in other studies. Future work should include tailored educational resources and behavioral interventions that can be provided to improve areas where patients feel less confident.

## AUTHOR CONTRIBUTIONS

**Alvina Acquaye‐Mallory:** Conceptualization (supporting); methodology (supporting); project administration (lead); visualization (supporting); writing – original draft (lead); writing – review and editing (equal). **Elizabeth Vera:** Conceptualization (supporting); data curation (lead); formal analysis (lead); methodology (supporting); visualization (supporting); writing – original draft (supporting); writing – review and editing (equal). **Anna Choi:** Conceptualization (supporting); methodology (supporting); project administration (supporting); writing – original draft (supporting); writing – review and editing (supporting). **Kathleen Wall:** Conceptualization (supporting); methodology (supporting); project administration (supporting); writing – original draft (supporting); writing – review and editing (supporting). **Mark R. Gilbert:** Conceptualization (supporting); methodology (supporting); writing – original draft (supporting); writing – review and editing (equal). **Terri S. Armstrong:** Conceptualization (lead); data curation (supporting); funding acquisition (lead); investigation (lead); methodology (lead); project administration (lead); resources (lead); supervision (lead); visualization (lead); writing – original draft (equal); writing – review and editing (equal).

## FUNDING INFORMATION

This research was supported in part by the Intramural Research Program of the National Institutes of Health, National Cancer Institute. The NOB Risk and Outcomes Study project is supported by Intramural Project 1ZIABC011768 (T.S.A.).

## CONFLICT OF INTEREST STATEMENT

All authors declare no conflict of interest.

## ETHICS STATEMENT

This study was performed in accordance with the ethical standards established by the US Federal Policy for the Protection of Human Subjects. The Institutional Review Board at the National Institutes of Health approved this study.

## CLINICAL TRIAL REGISTRATION NUMBER

ClinicalTrials.gov Identifier: NCT03251989.

## Supporting information


Table S1.

## Data Availability

The data that support the findings of this study are available on request from the corresponding author [TSA]. The data are not publicly available due to their containing information that could compromise the privacy of research participants.
