# Supplementary material for: Identifying associations between sample characteristics, symptoms, and self‐efficacy differences in adult patients with rare tumors of the central nervous system who participated in a novel web‐based natural history study
Source: Cancer Med. 2024 Aug 5;13(15):e70017. doi: 10.1002/cam4.70017 (PMC11299073; doi:10.1002/cam4.70017)
Supplement: Supplementary file 1 — Table S1. [file CAM4-13-e70017-s001.docx]

**Table S1. Sample Characteristics By Tumor Location**

|  |  |  |  |  |  |  |  |
| --- | --- | --- | --- | --- | --- | --- | --- |
|  | | |  | **Brain**  **N= 87** | | **Spine**  **N= 71** | |
|  | | |  | **n** | **%** | **n** | **%** |
| Sex | | | Female | 59 | 68 | 56 | 79 |
|  | | | Male | 27 | 31 | 14 | 20 |
|  | | | Non-binary/Third gender | 1 | 1 | 0 | 0 |
|  | | | Missing | 0 | 0 | 1 | 1 |
| Race | | | White | 83 | 95 | 67 | 94 |
|  | | | Other race | 3 | 3 | 4 | 6 |
|  | | | Missing | 1 | 2 | 0 | 0 |
| Hispanic/Latino | | | Yes | 4 | 5 | 3 | 4 |
|  | | | Missing | 1 | 1 | 0 | 0 |
| Marital status | | | Never married | 13 | 15 | 11 | 16 |
|  | | | Married | 56 | 64 | 46 | 65 |
|  | | | Divorced | 13 | 15 | 3 | 4 |
|  | | | Separated | 2 | 2 | 6 | 9 |
|  | | | Re-married | 1 | 1 | 2 | 3 |
|  | | | Widowed | 0 | 0 | 3 | 4 |
|  | | | Missing | 2 | 2 | 0 | 0 |
| Education | | | Some high school | 2 | 2 | 1 | 1 |
|  | | | High school graduate | 5 | 6 | 2 | 3 |
|  | | | Some college | 20 | 23 | 12 | 17 |
|  | | | College graduate | 32 | 37 | 31 | 44 |
|  | | | Any postgraduate work | 27 | 31 | 25 | 35 |
|  | | | 8th grade or less | 1 | 1 | 0 | 0 |
|  | | | Missing | 0 | 0 | 0 | 0 |
| Diagnosis | | | ATRT | 2 | 2 | 0 | 0 |
|  | | | Brainstem & Midline gliomas | 1 | 1 | 1 | 1 |
|  | | | Choroid plexus tumors | 1 | 1 | 0 | 0 |
|  | | | Ependymoma | 34 | 39 | 70 | 99 |
|  | | | Gliomatosis cerebri | 1 | 1 | 0 | 0 |
|  | | | Medulloblastoma | 4 | 5 | 0 | 0 |
|  | | | Oligodendrogliomas | 21 | 24 | 0 | 0 |
|  | | | Pineal region tumors | 20 | 23 | 0 | 0 |
|  | | | Pleomorphic xanthoastrocytomas | 3 | 3 | 0 | 0 |
| Treatment status | | | Newly diagnosed | 4 | 5 | 2 | 3 |
|  | | | No treatment other than surgery | 15 | 17 | 18 | 25 |
|  | | | On treatment | 19 | 22 | 5 | 7 |
|  | | | Follow-up without active treatment | 27 | 31 | 26 | 37 |
|  | | | Other | 20 | 23 | 18 | 25 |
|  | | | Missing | 2 | 2 | 2 | 3 |
| Time from symptoms | | | No symptoms | 8 | 9 | 1 | 1 |
| to surgery | | | <1 month | 10 | 11 | 3 | 4 |
|  | | | 1-2 months | 13 | 15 | 7 | 10 |
|  | | | 3-4 months | 9 | 10 | 5 | 7 |
|  | | | 5-6 months | 8 | 9 | 5 | 7 |
|  | | | 7-11 months | 7 | 8 | 14 | 20 |
|  | | | 1-4 years | 21 | 24 | 24 | 34 |
|  | | | >= 5 years | 4 | 5 | 10 | 14 |
|  | | | Missing | 7 | 8 | 2 | 3 |
| Recurrence | | | Yes | 14 | 16 | 13 | 18 |
|  | | | Missing | 10 | 11 | 2 | 3 |
| Work status | | | Not working | 44 | 51 | 30 | 43 |
|  | | | Working | 43 | 49 | 39 | 55 |
|  | | | Missing | 0 | 0 | 2 | 3 |
| Work changes | | | None | 30 | 34 | 25 | 35 |
|  | | | Reduce hours | 20 | 23 | 14 | 20 |
|  | | | Switch jobs | 2 | 2 | 2 | 3 |
|  | | | Retrain | 1 | 1 | 0 | 0 |
|  | | | Stop working | 27 | 31 | 23 | 33 |
|  | | | Lost work due to tumor | 6 | 7 | 5 | 7 |
|  | | | Missing | 1 | 1 | 2 | 3 |

**Table S2. Spearman Rho’s coefficients among Self-Efficacy in Managing Social, Managing Emotions, Overall symptoms, WAW, and REM**

|  | **Brain** | | **Spine** | |
| --- | --- | --- | --- | --- |
|  | **Social Interactions** | **Managing Emotions** | **Social Interactions** | **Managing Emotions** |
| **Overall symptom** | -0.55* | -0.54* | -0.04 | -0.25* |
| **WAW** | -0.43* | -0.40* | -0.03 | -0.15 |
| **REM** | -0.54* | -0.61* | 0.17 | -0.28* |
